# Accuracy of total knee arthroplasty using the modified gap technique based on the bone gap: an evaluation of the bone gap with a distal femoral trial component

**DOI:** 10.1186/s42836-021-00072-w

**Published:** 2021-04-05

**Authors:** Goki Kamei, Shigeki Ishibashi, Koki Yoshioka, Satoru Sakurai, Hiroyuki Inoue, Masakazu Ishikawa, Yu Mochizuki, Nobuo Adachi

**Affiliations:** 1 Department of Orthopaedic Surgery, Graduate School of Biomedical and Health Sciences, Hiroshima University, 1-2-3 Kasumi, Minami-ku, Hiroshima, Japan; 2 Department of Orthopaedic Surgery, Hiroshima Prefectural Hospital, 1-5-54, Ujinakanda, Minami-ku, Hiroshima, Japan; 3 Department of Artificial Joints and Biomaterials, Graduate School of Biomedical and Health Sciences, Hiroshima University, 1-2-3, Kasumi, Minami-ku, Hiroshima, Japan

**Keywords:** Modified gap technique, Joint gap size, Joint gap inclination, Distal femoral trial component

## Abstract

**Background:**

In total knee arthroplasty (TKA) using the modified gap technique, the soft-tissue balance is measured after osteotomy of the distal femur and proximal tibia (conventional bone gap). However, after osteotomy, the flexion gap size during 90° knee flexion may be larger than that observed after implantation. The tension of the lateral compartment during 90° flexion may also be reduced after osteotomy of the distal femur. We manufactured a distal femoral trial component to reproduce the condition after implantation and prior to posterior condyle osteotomy. This study aimed to evaluate the effect of the trial component on the flexion gap.

**Methods:**

This prospective study included 21 consecutive patients aged 78 years with medial osteoarthritis who underwent cruciate-retaining TKA between February 2017 and March 2018. The postoperative flexion gap size and inclination during 90° flexion were compared between cases with and without the trial component.

**Results:**

The mean joint gap size with the trial component (13.4 ± 0.80 mm) was significantly smaller than that without the trial component (14.7 ± 0.84 mm). The mean gap inclination angle with the trial component (3.7° ± 0.62°) was significantly smaller than that without the trial component (5.5° ± 0.78°).

**Conclusions:**

In the present study, the joint gap size and medial tension were significantly reduced after the trial component had been set. Accurate measurement of the soft-tissue balance is an important factor in the modified gap technique, and this method using a distal femoral trial component can offer better outcomes than those achieved with conventional methods.

## Introduction

In total knee arthroplasty (TKA) using the modified gap technique, the soft-tissue balance in extension and flexion is measured after osteotomy of the distal femur and proximal tibia, after which the rotation angle of the femur and the extent of bone resection from the posterior condyle are conventionally determined (conventional bone gap) [1–3].

**Fig. 1:**
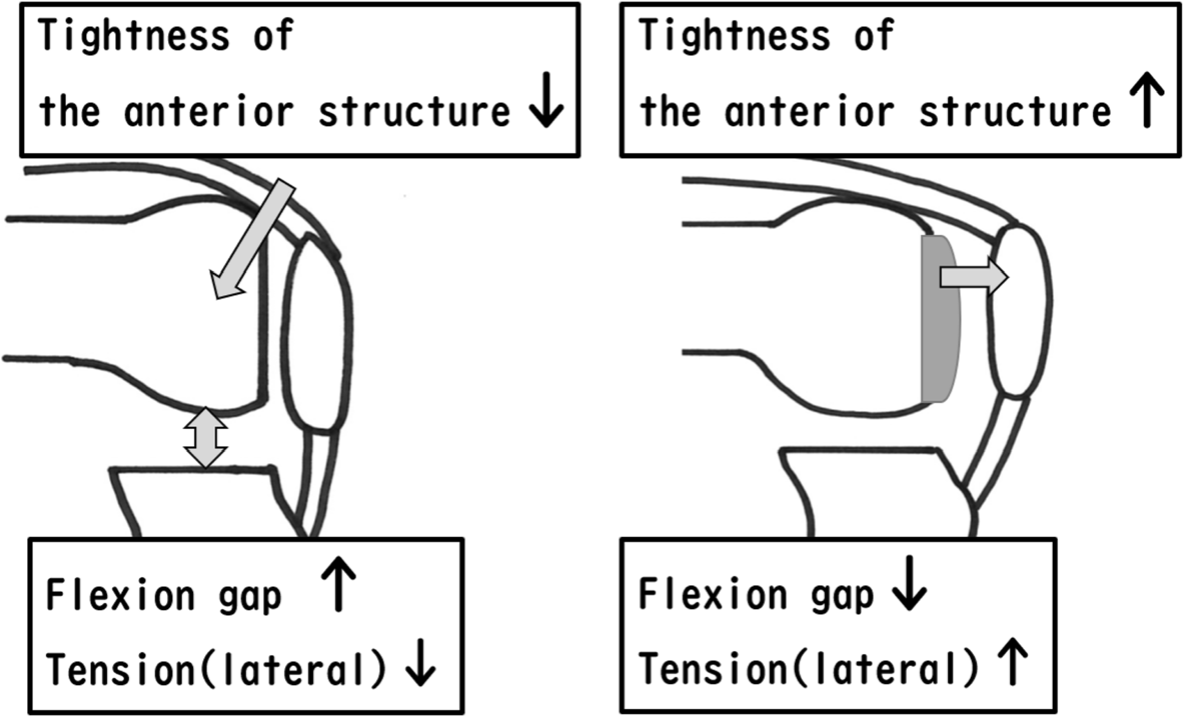
Distal femoral trial component

With the modified gap technique TKA, the basic concept is to achieve equal balance in both extension and 90°flexion. Intraoperative soft tissue balance has been reported to affect postoperative patient satisfaction and range of motion, and, therefore, it is important to measure the gap accurately [2, 4, 5]. However, conventional bone gap may not accurately reflect the balance of the gap after implant placement. After osteotomy, the flexion gap size during 90° knee flexion may be larger than that observed after implantation, since the extensor mechanism has been relaxed. The tension of the lateral compartment during 90° flexion may also be reduced after osteotomy of the distal femur (Fig. 1).

Therefore, we manufactured a distal femoral trial component (Zimmer-Biomet, Warsaw, Indiana, USA) to reproduce the condition after implantation and prior to posterior condyle osteotomy. This study aimed to evaluate the effect of the trial component on the flexion gap. We hypothesized that the flexion gap and joint gap inclination with the trial component would be smaller than those observed without the component.

## Materials and methods

### Patients and assessment

**Fig. 2:**
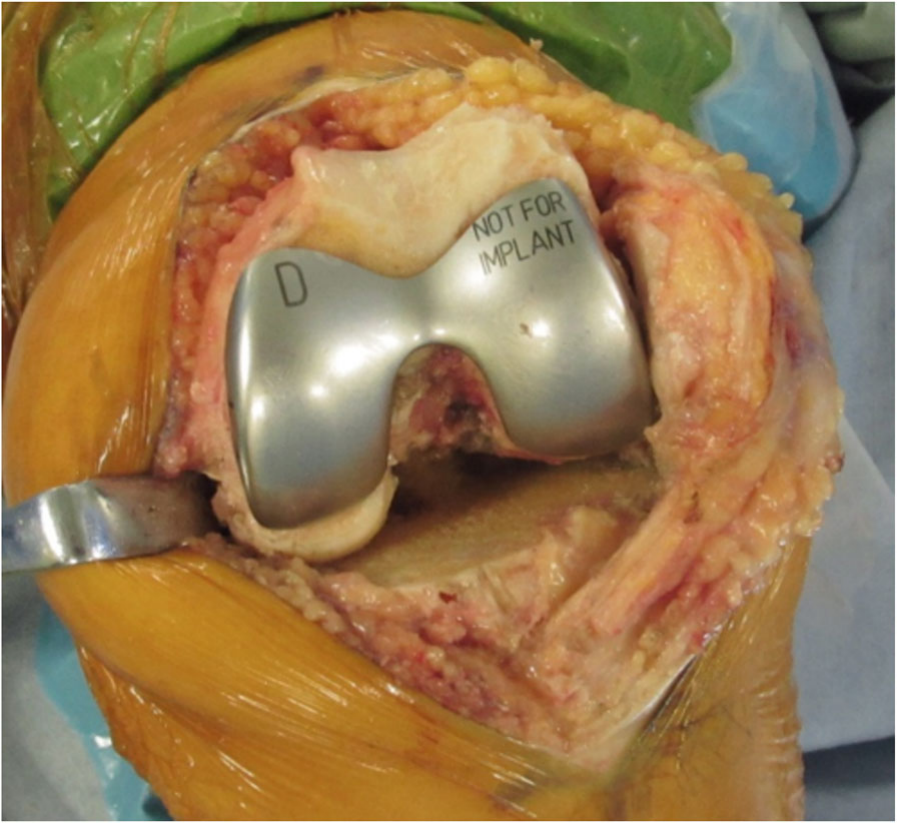
Intraoperative gap measurement without the trial component (left) and with the trial component (right)

This prospective study included consecutive patients with medial osteoarthritis who underwent Cruciate Retaining (CR)-TKA (Zimmer-Biomet’s Persona CR) using measured resection techniques between February 2017 and March 2018. Informed consent was obtained from all participants. Cases in which the gap was not measured were excluded. The patella was not resurfaced in all knees. A medial parapatellar approach was used in all cases. The distal femoral cut and proximal tibia cut were performed perpendicular to the mechanical axis. The trial component was equal to an implant (thickness: 9 mm) (Fig. 2). The flexion gap size and inclination during 90° flexion were measured using a JDK offset tensor (Stryker, Mahwah, NJ, USA) before and after setting the trial component and between cases with and without the trial component [6]. An attempt was made to minimize errors from creep elongation by applying 40 lb of tension and then actual measurement was taken by applying 30 lb of tension. When the medial compartment was tight, the gap inclination was positive. The posterior tibial slope angle was defined as the angle between the line perpendicular to the tibial axis and the posterior slope to the tibial plateau. The patellar height was assessed in terms of the Insall-Salvati ratio. All data were collected and analyzed retrospectively from an institutional-review-board-approved database (Hiroshima Prefectural Hospital), and the study was carried out according to the principles of the Declaration of Helsinki.

### Statistical analysis

The results were expressed as the mean ± standard deviation. Data were compared using the Wilcoxon test. The correlations between the flexion gap size and the posterior tibial slope angle and patellar height were assessed using Pearson correlation analysis. A *P* < 0.05 was considered statistically significant.

## Results

**Fig. 3:**
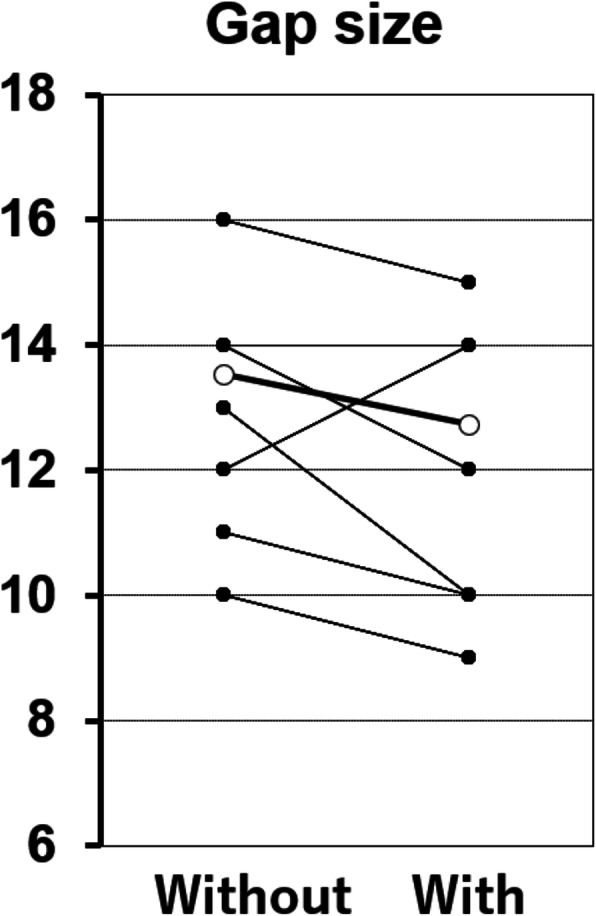
Gap size. The joint gap size during 90° flexion without (left) and with (right) the trial component. (○: mean gap size)

**Fig. 4:**
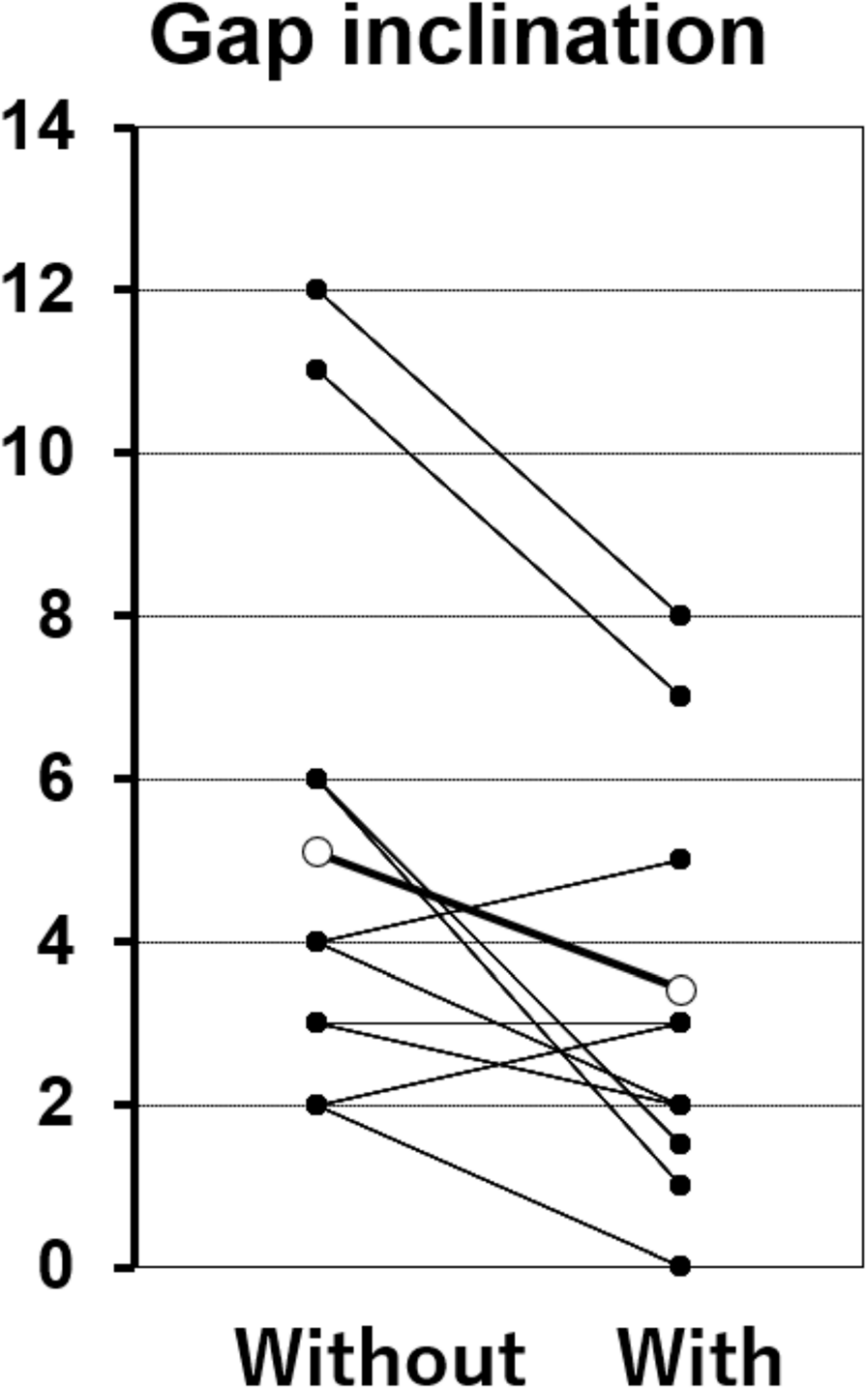
Gap inclination. The gap inclination during 90° flexion without (left) and with (right) the trial component. (○: mean gap size)

A total of 21 patients were included. There were five males and 16 females, and the mean age was 78 years. The mean joint gap size during 90° flexion was 14.7 ± 0.84 mm without the trial component and 13.4 ± 0.80 mm with the trial component. The mean gap inclination during 90° flexion was 5.5° ± 0.78°  without the trial component and 3.7° ± 0.62° with the trial component. The joint gap size and joint gap inclination were both significantly lower with the trial component than without the trial component (*P* = 0.0016, *P* = 0.0012, respectively) (Figs. 3 and 4).

**Fig. 5:**
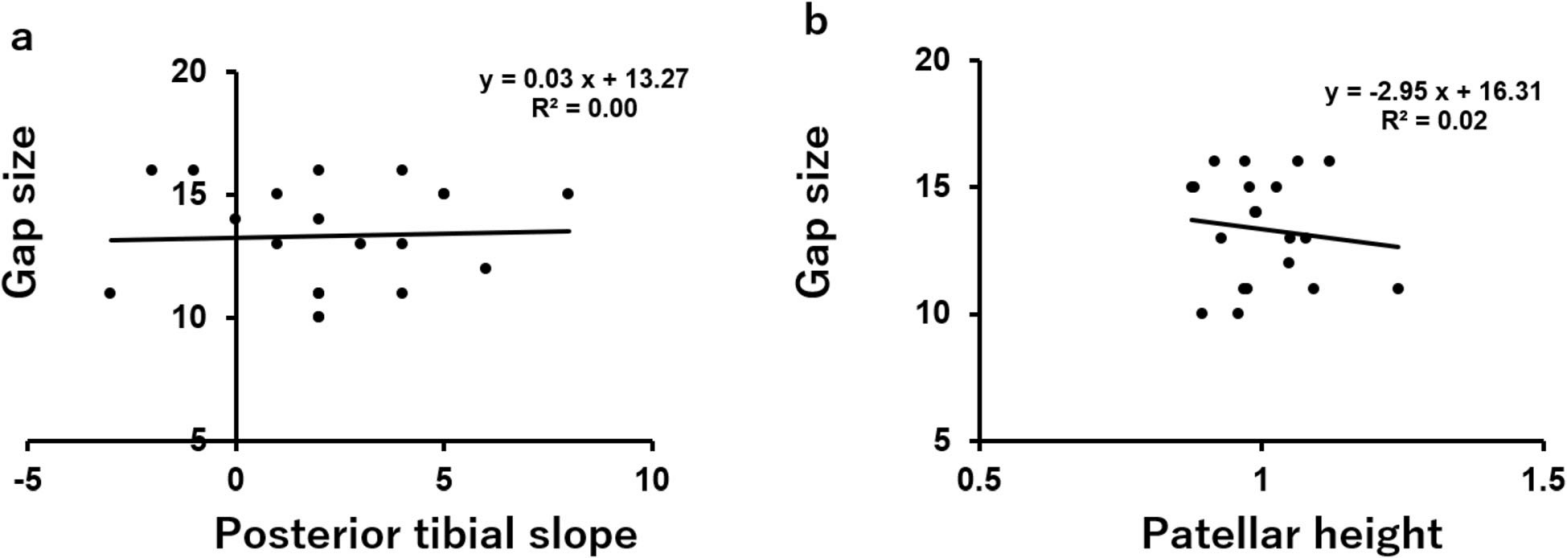
The correlation between the flexion gap without the trial component and posterior tibial slope angle (**a**) or patellar height (**b**)

**Fig. 6:**
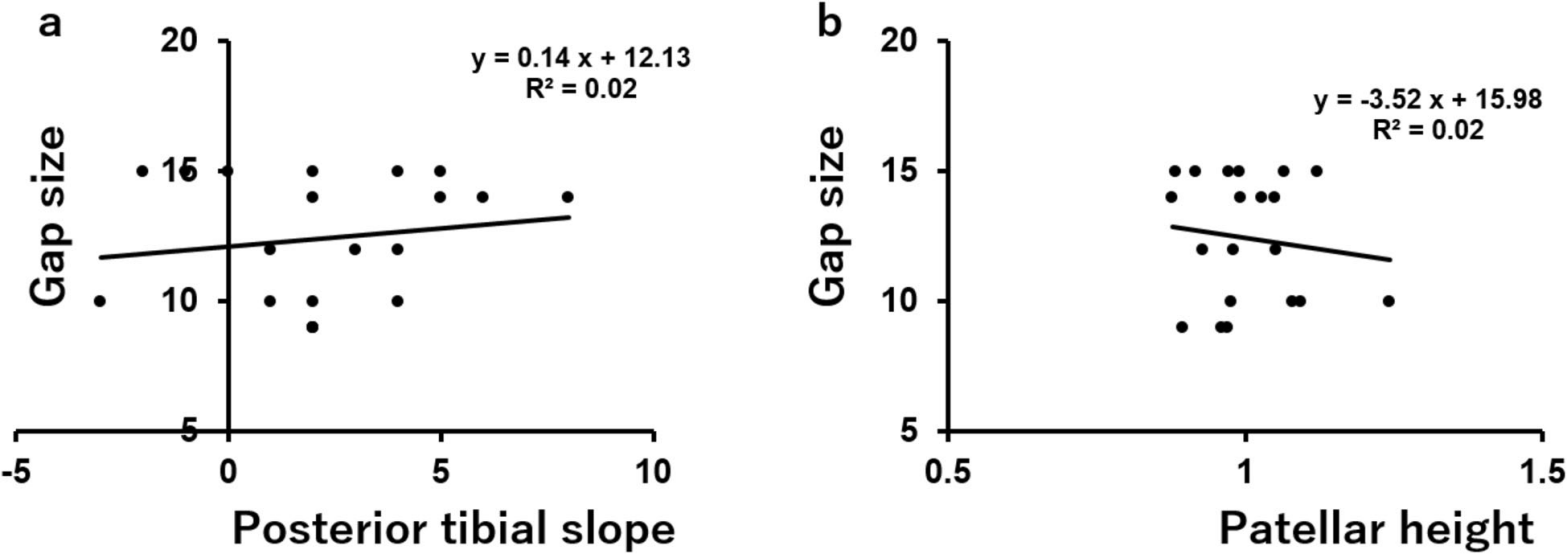
The correlation between the flexion gap with the trial component posterior tibial slope (**a**) or patellar height (**b**)

There was no correlation between the flexion gap without the trial component and posterior tibial slope angle (*r* = 0.0418, *P* = 0.4305) (Fig. 5a) or patellar height (*r* = 0.1249, *P* = 0.2998) (Fig. 5b). Similarly, there was no correlation between the flexion gap with the trial component and posterior tibial slope angle (*r* = 0.1545, *P* = 0.2577) (Fig. 6a) or patellar height (*r* = 0.1327, *P* = 0.2886) (Fig. 6b).

## Discussion

In TKA using the modified gap technique, the soft-tissue balance is typically measured after osteotomy of the distal femur and proximal tibia. The implant position, rotation angle of the femoral implant, and extent of bone resection from the posterior condyle are then determined [7–9]. This is the most important factor for accurately evaluating the joint gap size and inclination in TKA using the modified gap technique [10–12]. However, because an implant is not present in the distal femur, the tension of the forward soft tissue is relaxed when the joint gap is measured, and the flexion gap might, as a result, be larger postoperatively than during implant setting. Furthermore, because the tension on the lateral side is reduced when the joint gap is measured, the gap inclination may be larger after than during implant setting.

It has been reported that posterior cruciate ligament (PCL) resection, the posterior tibial slope, the patellar height, and the setting of a trial component affect the knee flexion gap in TKA [13–19]. Kadoya *et al*. reported that the flexion gap was 2 mm smaller than the extension gap prior to PCL resection, and 1.7 mm larger afterwards [13]. Oka *et al*. reported that the joint component gap during 90° flexion was positively correlated with the tibial slope in posterior stabilized (PS) TKA [14]. Okazaki *et al*. showed that  changing the tibial slope by 5° altered the flexion gap by approximately 2 mm with CR-TKA and 1 mm with PS-TKA [15]. Nishizawa *et al*. reported that the patellar height influenced the soft-tissue balance and was positively correlated with the joint component gap at greater flexion angles (90° and 135°) in PS-TKA, but the PCL was an important factor influencing the flexion soft-tissue balance in CR-TKA [16]. In the present study, there was no significant correlation between the flexion gap (with and without the trial component) and the posterior tibial slope angle or patellar height. Because this study was not a comparison of cases with a difference of more than 5 degrees as reported by Okazaki *et al*. [15], we did not find a correlation between the flexion gap with or without the trial component and posterior tibial slope. Moreover, this study focused only on CR TKA to exclude the influence of PCL resection on the flexion gap. Since PCL seems to play an important role in the flexion gap, the correlation with posterior tibial slope with and without the trial component in PS-TKA will be examined in the future.

Hananouchi *et al*. reported that the intraoperative gap difference between flexion and extension was significantly greater with the use of a trial component than without the component. This difference was attributed to the surrounding soft-tissue tightness or elasticity [17]. Hayashi *et al*. reported that the intraoperative component gap kinematics was affected by the tightness of the posterior structure during extension and the tightness of the anterior structure during deep knee flexion [18]. The results of Hananouchi and Hayashi suggest that it is very important to recreate the condition after placement of the component prior to excision of the posterior condyle. The present study is of great importance for the case of modified gap TKA, because it is paramount to accurately assess the gap and perform the surgery.

In conventional TKA using the modified gap technique, the flexion bone gap is typically measured during a state of relaxed forward tension, after which the posterior condyle is cut with reference to the bone gap. Therefore, the quantity of bone resected in the osteotomy is smaller than necessary, and a larger component is used. Consequently, the posterior condyle of the component tightens the posterior structures, and the joint gap during extension is reduced [19, 20]. The present study demonstrated that the medial tension during 90° flexion was significantly reduced after implantation of the trial component. Hence, in the conventional method, the femoral component is likely set in a position with excessive external rotation. The posterolateral condyle offset is therefore increased, and the posterolateral condyle of the component tightens the posterior structures [21]. In addition, this setting in a position with excessive external rotation reduces the joint gap during extension. The exact soft-tissue balance is not measured during either extension or flexion in conventional TKA using the modified gap technique.

In the present study, by using a distal femoral trial component, the joint gap size and medial tension was significantly reduced after the trial component had been set. Accurate measurement of the soft-tissue balance is an important factor in the modified gap technique, and this method using a distal femoral trial component may yield better outcomes than those attained with conventional methods.

The limitations of this study were that perioperative measurements were not reflected in the surgical procedure. The current study evaluated the intraoperative gap difference with and without a distal femoral trial component. The femoral component was placed based on these results and the feel of the spacer block insertion. Therefore, the joint gap observed with the use of the distal femoral trial component could not be compared to the component gap.

## Conclusions

In the present study, the joint gap size and medial tension were significantly reduced after the trial component had been set. Accurate measurement of the soft-tissue balance is an important factor in the modified gap technique, and we can accomplish better outcomes by using a distal femoral trial component than those obtained with conventional methods. In future, we will further evaluate the gap difference between PS-TKA with and without a distal trial component, and will compare the joint gap observed with the use of the distal femoral trial component to the component gap in the cases in which the surgery is performed with reference to the joint gap with the distal femoral trial component.

## Data Availability

All data generated or analyzed during this study are included in this published articles.

## Abbreviations

TKA: Total knee arthroplasty
CR: Cruciate retaining
PCL: Posterior cruciate ligament
PS: Posterior Stabilized
